# High Fibrinogen in Peripheral Blood Correlates with Poorer Hearing Recovery in Idiopathic Sudden Sensorineural Hearing Loss

**DOI:** 10.1371/journal.pone.0104680

**Published:** 2014-08-28

**Authors:** Sho Kanzaki, Masafumi Sakagami, Hiroshi Hosoi, Shingo Murakami, Kaoru Ogawa

**Affiliations:** 1 Department of Otorhinolaryngology, Head and Neck Surgery, Keio University, School of Medicine, Shinjuku, Tokyo, Japan; 2 Department of Otorhinolaryngology, Hyogo College of Medicine, Nishinomiya, Hyogo, Japan; 3 Department of Otorhinolaryngology, Nara Medical University, Kashihara, Nara, Japan; 4 Department of Otorhinolaryngology, Graduate School of Medical Sciences, Nagoya City University, Mizuho, Nagoya, Aichi, Japan; Harvard University, United States of America

## Abstract

**Objectives:**

We used hearing tests and peripheral blood sample analyses to characterize the pathology of idiopathic sudden sensorineural hearing loss (ISSNHL) and to identify possible prognostic factors for predicting recovery of hearing loss.

**Study Design:**

A retrospective, multicenter trial was conducted.

**Methods:**

Two hundred three patients examined within 7 days after the onset of ISSNHL received prednisone with lipo-prostaglandin E1. Pure-tone auditory tests were performed before and after treatment with these drugs. Blood tests were performed on blood samples collected during the patients’ initial visit to our clinic.

**Results:**

In all patients, elevated white blood cell (WBC) counts, fasting blood sugar levels, HgbA1c, and erythrocyte sedimentation rate (ESR) significantly correlated with high hearing threshold measurements obtained on the initial visit. High fibrinogen levels, WBC counts, ESR, and low concentrations of fibrinogen degradation products (FDP) were associated with lower hearing recovery rates. Additionally, different audiogram shapes correlated with different blood test factors, indicating that different pathologies were involved.

**Conclusions:**

High fibrinogen levels measured within seven days after ISSNHL onset correlated with poorer hearing recovery. This may be a consequence of ischemia or infections in the inner ear. The high WBC counts also observed may therefore reflect an immune response to inner ear damage induced by ischemic changes or infections. Our data indicate that therapeutic strategies should be selected based on the timing of initial treatment relative to ISSNHL onset.

## Introduction

Idiopathic sudden sensorineural hearing loss (ISSNHL) is characterized by a sudden onset of hearing loss and generally presents unilaterally [1]. Little is known about the pathogenesis of ISSNHL. However, a diversity of treatments has been proposed to cover all the possible underlying mechanisms of the disease, such as those related to viruses, the immune system, and vascular injury [2], [3].

Many studies have identified factors that predict which patients possess a favorable prognosis and require minimal or no treatment. However, only a few studies have determined the probability of whether a patient’s hearing will recover by using prognostic models that incorporate probability data [4]. Several reports have investigated the prognostic value of certain factors, such as the timing of initial treatment, initial hearing levels, absence or presence of accompanying vertigo, and audiogram profiles [4], [5], [6], [7], [8]. Beginning treatment earlier, soon after diagnosis results in a better prognosis and decreases patients’ likelihood of acquiring irreversible damage from inner ear pathologies. Audiogram profiles may also indicate different pathologies that lead to cochlear lesions as well as different prognoses.

Other researchers have tested various blood indices as potential prognostic factors for ISSNHL. Analyses of peripheral blood samples from patients who experience ISSNHL revealed they present with certain vascular risk factors, such as elevated plasma and whole blood viscosity [9]_,_ [10], prothrombotic genes [11], [12], [13], [14], and elevated fibrinogen levels [3], [15]. This suggests that these factors are involved in the pathology of ISSNHL. As these factors were not correlated with hearing recovery, we did not consider them as candidates for potential prognostic factors.

It is well known that vascular risk factors can affect inner ear function by compromising the vascular endothelium, leading to functional disruption [11]. Studies indicate that ISSNHL patients have higher levels of circulating adhesion molecules [16], [17]. Other studies on immunologic factors in ISSNHL show that anti-heat shock protein (HSP) 70 autoantibodies correlate well with a good prognosis [18]. Antinuclear antibody (ANA) titer and erythrocyte sedimentation rate (ESR) are two other blood parameters that are significantly elevated in ISSNHL patients compared to normal individuals [19].

White blood cell (WBC) count is another potential prognostic factor. High WBC counts are frequently found in ISSNHL patients [20]. Guided by cytokines and other soluble molecules, WBCs migrate to vascular epithelial cells [16]. WBCs are usually involved in tissue damage after cardiac ischemic changes [21] and stroke [22]. Thus, they may similarly contribute to the inner ear tissue damage seen in ISSNHL.

In this study, we performed hearing tests and analyzed peripheral blood samples to characterize the pathology of ISSNHL and to identify prognostic factors for predicting hearing recovery.

## Subjects and Methods

Subjects were 203 patients with unilateral ISSNHL (Table 1), who were seen at different hospitals between January 2005 and May 2012. The hospitals were all registered to participate in the present study. This study, which involved using retrospective data, was approved by the ethical committees of Keio University School of Medicine, Hyogo College of Medicine, Nara Medical University, and Nagoya City University (International Clinical Trials Registry JPRN-UMIN000008356). No new data were obtained for this study. The patients consented that their data could be used for future studies. The authors were involved in the initial collection of the data. The university hospitals stated in the original, written informed consent that any patient data obtained initially could be used in future studies. Data was anonymized at the time of collection.

**Table 1:** Diagnostic criteria for idiopathic sudden sensorineural hearing loss (ISSNHL)*.

**Main symptoms**	
1	Sudden onset of hearing loss
2	Sensorineural hearing loss, usually severe
3	Unknown cause
**Accessory symptoms**	
1	May be accompanied by tinnitus
2	May be accompanied by vertigo, nausea, and/or vomiting without recurrent episodes
3	No cranial nerve symptoms (other than from cranial nerve XIII)

*ISSNHL was defined according to criteria described by the Sudden Deafness Research Committee of the Japanese Ministry of Health and Welfare [23]. Patients were diagnosed with *definite ISSNHL* if they presented with all the listed symptoms, or with *probable ISSNHL* if they presented mainly with only symptoms 1 and 2.

**Figure 1:**
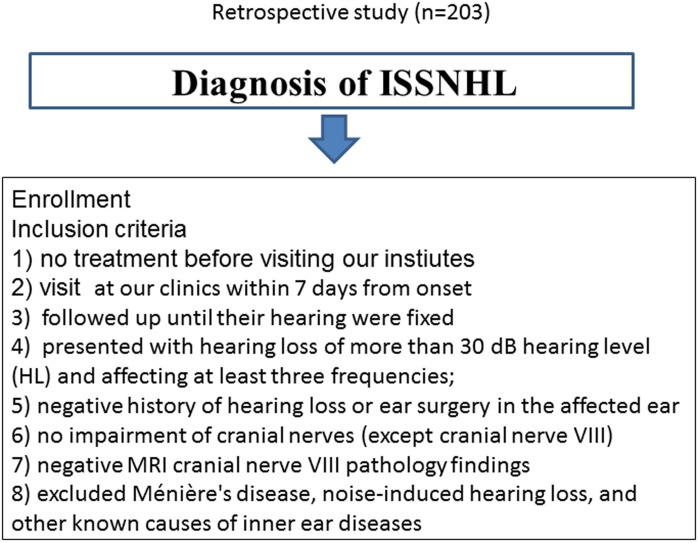
Flowchart showing patient inclusion criteria.

The study was conducted according to the principles expressed in the Declaration of Helsinki. Inclusion criteria were as follows: (1) hearing loss appeared acutely, without recognizable cause; (2) hearing loss was sudden, sensorineural in origin, and untreated; (3) patients were seen at our clinics within 7 days of onset; (4) patients followed up until their hearing recovered; (5) patients presenting with hearing loss of more than a 30 dB hearing level (HL), affecting at least three frequencies; (6) normal hearing in the contralateral ear (air conduction pure-tone average [PTA] at 0.25, 0.5, 1, 2, 4, and 8 kHz frequencies were <40 dB HL); (7) negative history of hearing loss or ear surgery in the affected ear; (8) lack of impairment of the cranial nerves (except cranial nerve VIII); and (9) gadolinium-enhanced magnetic resonance image verifying negative cranial nerve VIII pathology. All patients affected by Ménière’s disease, noise-induced hearing loss, and other known causes of inner ear disease (including viral infection) were excluded (Figure 1) (Protocol S1).

On the patients’ initial visit to one of our clinics, a complete clinical history was obtained and standard audiovestibular exams were performed. These consisted of pure-tone testing and MRI of the internal auditory canal and posterior cranial fossa. Peripheral blood samples for chemistry and hematological tests were collected from the patients after informed consent was obtained but before treatment was given. All patients were then subjected to the same treatment protocol, which consisted of intravenous prednisone (60 mg tapered to 20 mg), Lipo PGE1 (vasodilators), ATP, and vitamin B12 for 7 days. Hearing improvement was evaluated using the following improvement rate criterion described by Ogawa et al. [23]. Soon after the patients’ hearing stabilized, recovery was evaluated as follows:
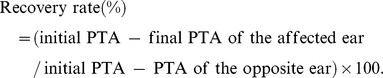



Blood samples were analyzed. Clinical chemistry determinations were made for glucose, hemoglobin A1c (Hgb A1c), total cholesterol, high-density lipoprotein (HDL), low-density lipoprotein (LDL), and serum triglyceride levels. Hematology determinations were made for WBCs, red blood cells (RBCs), thrombocytes, hemoglobin, hematocrit, and ESR. Hemostasis determinations were made for prothrombin time (PT %), activated partial thromboplastin time (APTT %), fibrinogen, and fibrinogen degradation products (FDPs).

We examined whether initial hearing or hearing recovery rate correlated with the above-mentioned blood data values. All results were expressed as mean values ± standard deviation. Statistical analysis was performed using parametric and nonparametric tests (SPSS 17.0, Chicago, IL, USA).

## Results

The data obtained from patients with ISSNHL were summarized and tabulated. Table 2–6 presents the patients’ clinical characteristics. There were no significant differences in the prognoses of male and female subjects (Table 2). Total deafness and having a high-pitch, sloping audiogram shape translated to poorer recovery rate (Table 3). On average, blood test data were not abnormal (Table 4). Subjects with vertigo exhibited a poorer hearing recovery rate. Only 13 patients with tinnitus, ear fullness, and vertigo had a poor hearing recovery rate (Table 5). Time of initial treatment relative to ISSNHL onset (1–7 days) did not correlate with hearing recovery rate (Table 6).

**Table 2:** Gender and hearing recovery rate.

Gender	No. of cases	Hearing recovery rate	HL at initial visit (dB)
**Male**	87	70.1%	67.99
**Female**	116	58.4%	67.94
**Total**	203	63.4%	67.96

**Table 3:** Audiogram shape and hearing recovery rate.

Audiogram shape	No. of cases	Recovery rate (%)	Hearing level at initial exam (dB)
**Flat**	48	62.5%	73.21
**High**	35	48.5%	50.49
**Total**	31	54.7%	99.71
**Low and high**	8	65.6%	48.63
**Mid**	25	82.2%	63.00
**Other**	53	69.4%	61.62
**Unknown**	3	73.2%	64.67

**Table 4:** Average hearing level data and laboratory blood test values.

	Average	Standard deviation
*Initiation of treatment relative to ISSNHL onset (days)	2.3	1.8
Initial hearing (dB)	68.0	23.0
Hearing recovery rate (%)	63.4	56.5
WBC	6.6	2.1
RBC	4.6	0.5
Plt	224.7	52.6
Hct (%)	40.9	4.6
PT (%)	19.9	25.8
APTT (%)	26.8	4.1
Fibrinogen	286.8	55.5
FDP	5.4	17.0
FBS	119.7	38.0
HgbA1c	5.6	1.3
Total cholesterol	214.6	35.7
LDL	129.1	30.9
TG	119.4	85.3
ESR	13.1	14.2

**Table 5:** Correlation of symptoms with initial hearing levels and recovery rate.

	No. of cases	Recovery rate (%)	Hearing level at initial exam (dB)
**Tinnitus**	126	60.7%	67.60
**Vertigo**	62	46.2%	74.95
**Fullness**	81	63.2%	64.52
**Tinnitus and vertigo**	24	43.6%	75.00
**Tinnitus, vertigo, and fullness**	13	33.2%	77.08
**Vertigo and fullness**	7	47.9%	60.14
**Tinnitus and fullness**	34	67.5%	64.65

**Table 6:** Treatment timing and hearing recovery rate.

Timing (days)*	No. of cases	Recovery rate (%)	Hearing level at initial exam (dB)
0	28	63.6%	78.69
1	55	70.1%	69.49
2	40	65.4%	67.33
3	35	68.7%	63.26
4	16	49.9%	69.81
5	12	56.9%	61.25
6	11	34.5%	60.45
7	6	59.5%	57.83

APTT, activated partial thromboplastin time; ESR, erythrocyte sedimentation rate; FBS, fasting blood sugar; FDP, fibrinogen degradation products; Hct, hematocrit; HgbA1c, hemoglobin A1c; LDL, low-density lipoprotein cholesterol; Plt, platelets; PT, prothrombin time; RBC, red blood cell count; TG, triglycerides; WBC, white blood cell count.

*Initiation of treatment relative to ISSNHL onset; zero day is baseline value.

High WBC counts 1–7 days after the onset of sensorineural hearing loss correlated with hearing recovery rate (Table 7). In subjects who received treatment within seven days of ISSNHL onset, hearing recovery rate correlated with high WBC counts, elevated fibrinogen levels, and elevated ESR (Table 8). Taken together, our data indicate that hearing recovery rate was correlated with the patients’ hearing and fibrinogen levels at the time they first sought hospital treatment for their hearing loss (i.e., 1–7 days after onset).

**Table 7:** Correlation between patient hearing levels measured during the initial exam with blood test values of patients treated within 7 days of ISSNHL onset.

	R	P value
WBC	0.166	0.019*
FBS	0.255	0.001**
HgbA1c	0.168	0.039*
ESR	0.307	0.005**

WBC, white blood cell count; FBS, fasting blood sugar; ESR, erythrocyte sedimentation; rate; R, rate.

*p<0.05; **p<0.01.

**Table 8:** Correlation between patient hearing recovery rate and blood test values of patients treated within 7 days of ISSHNL onset.

	R	P value
WBC	−0.202	0.005**
Fibrinogen	−0.264	0.004**
FDP	0.204	0.029*
ESR	−0.259	0.020*
Initial hearing	−0.226	0.001**

WBC, white blood cell count; ESR, erythrocyte sedimentation rate; FDP, fibrinogen degradation products; R, rate.

*p<0.05; ** p<0.01.

We also classified several types of audiogram shapes and analyzed their correlation with prognostic factors (see Table 9). We used simple linear regression analysis, with fibrinogen as the only factor (RR = 146.376–0.270 * fibrinogen (p = 0.026) (R = 0.274, R^2^ = 0.075, Adjusted R^2^ = 0.060). Multivariate analyses (multiple regression) failed to identify any relevant factors.

**Table 9:** Correlation between various potential prognostic blood factors and audiogram shape and hearing recovery rate.

Audiogram shape	Hearing recovery rate		
	Factors	R	P value
**Flat**	None	–	–
**High-pitch sloping**	Fibrinogen	−0.406	0.04
	ESR	−0.639	0.01
**Total deafness**	WBC	−0.397	0.03
	FBS	−0.447	0.03
	LDL	−0.655	0.02
	Hct	+0.661	0.00
**Low and high**	None	–	–
**Middle-pitch notch**	Fibrinogen	−0.625	0.01
	TG	+0.563	0.01
**Other**	WBC	−0.312	0.02

+, Positive correlation; –, negative correlation; ESR, erythrocyte sedimentation rate; FBS, fasting blood sugar; Hct, hematocrit; LDL, low-density lipoprotein cholesterol; Plt, platelets; PT, prothrombin time; TG, triglycerides; WBC, white blood cell count.

## Discussion

Here, we report results from an ongoing multicenter clinical trial evaluating prognostic factors for ISSNHL. In all 203 patients included in this study, elevated fibrinogen levels and WBC counts correlated with poorer hearing recovery rates and poorer prognosis. Higher ESR values and WBC counts obtained during the patients’ initial hospital visit significantly correlated with higher hearing thresholds (average threshold measured at five frequencies).

It remains unclear whether ESR is a reliable prognostic factor for ISSNHL, as some researchers consider it to convey a poor prognosis [2], while others consider it to have no prognostic value at all [7]. In the present study, elevated ESR values obtained 1–7 days after disease onset correlated not only with the patients’ initial hearing levels but also with disease outcome in ISSNHL. Indeed, ESR values correlated positively with fibrinogen levels (r = 0.435, P = 0.0001). We speculate that increased levels of fibrinogen increase ESR; thus, the elevated ESR observed in our patients may represent a secondary reaction to fibrinogen. Elevated fibrinogen and ESR values occur in response to inflammation, tissue damage, viral infection, autoimmune disease, and ischemic changes [24].

As previously reported, ANA titer and ESR are two parameters that are significantly more elevated in ISSNHL patients than in normal individuals [19]. Nevertheless, the significance of these parameters remains controversial [19].

The elevated WBC counts in our patients may be due to an immune response to more extensive tissue damage in the inner ear. This hypothesis is consistent with our finding that higher WBC counts negatively correlates with hearing recovery rates in patients having a “total deafness” audiogram shape and in patients with severe hearing loss (Table 9).

Higher WBC counts did correlate with hearing level measured during the initial visit and hearing recovery of patients that sought treatment within 1–7 days after onset (Tables 7 and 8). Myocardial infarct size has been shown to be directly correlated with increased WBC counts [25]. The same may also apply to cases involving inner ear damage.

As mentioned, within 1–7 days of ISSNHL onset, fibrinogen levels correlate negatively with hearing recovery rate, and elevated ESR correlates with the patients’ hearing level at the initial visit. The finding that fibrinogen levels measured one day after ISSNHL onset correlates with hearing recovery rate indicates that, at ISSNHL onset, blood viscosity is high and thus blood supply to affected areas is decreased. We surmised that a few days thereafter, inner ear tissue damage further progresses due to WBCs being drawn to the site by adhesion molecules expressed by local blood vessels.

From the start of this study in 2005, we have used vasodilators to treat ISSHL, even though their use for this purpose remains unsubstantiated due to the poor hearing outcome. However, one of our co-authors (K. Ogawa) has observed marked hearing improvement at higher frequencies and significant improvement of tinnitus in patients receiving PGE1 compared to placebo (44% versus 13%), even though no significant differences were observed in the overall hearing gain or in the rate of hearing improvement between the PGE1 and placebo groups of patients [23]. Nevertheless, we chose to treat our ISSHL patients with the vasodilator lipoPGE1, even though sufficient evidence supporting its use in this patient population is lacking at this time.

The time course of molecular factors, such as adhesion molecules, contributing to inner ear damage might be influenced by transcriptional factors. Indeed, nuclear factor kappa B (NFkB), a transcriptional factor that activates cytokines and adhesion molecules, may be involved in inner ear damage or sensorineural hearing loss [26]. In animal models of inner ear damage, such as acoustic trauma, NFkB and cytokines have been shown to promote inner ear damage just a few hours after the delivery of acoustic trauma (noise) [27], [28]. Macrophages migrate into the cochlea a few days later [29].

We also demonstrated that audiogram shape varies across different pathological backgrounds that require different prognostic factors for blood tests.

Our data demonstrate that patients with higher LDL values have lower hearing recovery rates. High LDL and high cholesterol levels increase atherosclerosis and blood viscosity. Cholesterol, however, is not prognostic factor for ISSHL. This may induce extensive inner ear ischemia, which could be responsible for the “total deafness” audiogram pattern observed in some of our patients.

Canis et al. recommended fibrinogen and LDL apheresis in patients presenting with high fibrinogen and LDL levels [30]. Our data demonstrate that apheresis treatment may be indicated in patients with middle-pitch notch, higher-pitch sloping, or total deafness audiogram shapes.

In the present study, we propose a novel therapeutic concept for the treatment of ISSNHL, in which treatment choice depends on the timing and type of audiogram obtained.

We demonstrated that high fibrinogen levels (measured one day after ISSNHL onset) correlate with poorer hearing recovery in ISSNHL. High fibrinogen levels may indicate ischemic changes in the inner ear [30]. High WBC counts may reflect an immune response to inner ear damage induced by ischemic changes or infections. Thus, elevated WBC counts suggest that several causative factors may contribute to ISSNHL pathology. Our data have also led us to conclude that the time course of ISSNHL (i.e., progression of tissue damage after onset) and audiogram shape dictate different therapeutic strategies. Additional prospective studies are necessary to further elucidate the precise mechanisms underlying ISSNHL pathology.

## Supporting Information

Table S1 **Data of all the patients in this study.**
(XLSX)

Checklist S1 **TREND checklist.**
(PDF)

Protocol S1 **We provided the protocol (English version and Japanese original version).**
(DOCX)
