# Supplementary material for: High Fibrinogen in Peripheral Blood Correlates with Poorer Hearing Recovery in Idiopathic Sudden Sensorineural Hearing Loss
Source: PLoS One. 2014 Aug 28;9(8):e104680. doi: 10.1371/journal.pone.0104680 (PMC4148242; doi:10.1371/journal.pone.0104680)
Supplement: Protocol S1 — We provided the protocol (English version and Japanese original version). (DOCX) [file pone.0104680.s003.docx]

Protocol of Project

1. **Objects and Methods**

**Background:** Blood tests were performed at the first visit before treatment of steroid and also hearing tests were examined before and after treatment.

- Blood tests

leukocytes, erythrocytes, thrombocytes, hemoglobin, hematocrit, prothrombin time ( PT%), activated partial thromboplastin time (APTT％)**,**fibrinogen and fibrinogen degradation products (FDPs). **HbA1c** 、glucose, total cholesterol, high-density lipoprotein (HDL), low-density lipoprotein (LDL), and triglyceride levels, and ESR.

- Pure tone audiometry, average of 5 frequencies

**Objectives:** The etiology of I idiopathic sudden sensorineural hearing loss (ISSNHL)is unknown. In this study, we used hearing tests and blood sample analyses to analyze the correlations between blood tests and hearing recovery between before and after treatment and to characterize both the pathology of ISSNHL, and possible prognostic factors for predicting recovery of hearing loss.

**Methods:** The subjects were patients of several hospitals between January 2005 and May 2012. Totally, more than two hundred　patients seen within 7 days after the onset of ISSNHL received prednisone with lipo-prostaglandin E1.

The Pure tone audiometry was performed at the first visit and at the time when their hearing was fixed hearing loss. Blood tests were performed on blood samples collected during the patients’ first visit.

We retrospective study to observe the correlation between the hearing in patients who performed blood tests (before treatment) at the first visit of ISSHL patients who were registered from January 2005 to May 2012.

2 Number of subjects
Total cases (All of the subjects in the Keio University, the Hyogo College of Medicine, the Nara Medical University, and the Nagoya City University) 200 cases or more
Keio University Hospital ; over 80

3 implementation period
from January2005 to May 31, 2012

4 implementation site
We collected data from patients with ISSHL in the Keio University, the Hyogo College of Medicine, the Nara Medical University, and the Nagoya City University analyzed department of at the laboratory Otolaryngology at the Keio University.

**Selection of Criteria and details on cooperation**

**1 Selection of criteria**Pure tone audiometry was performed prior to the initiation of therapy, (2 months after normal when the fixed time, and does not improve to improve if the hearing has improved immediately) cases month course has finished or two or hearing when fixed blood test is course is observed up. This is a multicenter study (shown in Appendix diagnostic criteria for ISSSHL, on the basis of the diagnostic criteria, it is analyzed only for ISSHL reliably example variations so as not to cause the diagnosis.

**2 Requirements to the patients**

Since however a retrospective study, notification is not to use the patient for analysis. We have explained verbally the necessity of blood test.
We are currently a web publication, such as the following in our department as public information.

**Request for cooperation in clinical research**In our clinic, we asked in clinical research as below, to help medical future test results of various recorded at the time of practice, as valuable data to publish their research and conference presentation it is possible that I am allowed to use. With the consent of the Keio University School of Medicine Ethics Committee, will be sufficient consideration to anonymous and personal information. If there is any questions, please ask your doctors.
**Research Overview and the Principal Investigator and**
· Clinical research for diagnosis and treatment related to ear disease, such as hearing loss, tinnitus Including the development of new testing and treatment.

Principal Investigator: Sho Kanzaki (and Professor Kaoru Ogawa)
Contact: Dept. Otolaryngology, School of Medicine, Keio University Otolaryngology
Phone 03-1211 -3353

We followed clinical research in accordance with clinical guidelines.
①Significance purpose, and method of the research ; we analyzed hearing prognosis and blood test in patients with ISSHL to perform the pathogenesis and prognostic factors.

We try to detect etiology, bio marker of hearing prognosis. We also analyze the data of the hearing test and blood tests.
② Research Institution
Dept. Otolaryngology, the Keio University, the Hyogo College of Medicine, the Nara Medical University, and the Nagoya City University
③ We protect their personal information in accordance with clinical guidelines

④　We support appropriately and promptly complaints and inquiries from the subject concerning the handling of personal information related to the research.
⑤　When the patients required to disclose their own personal information, we disclose personal information.

3. Details of the cooperation ; permission of use of medical records and blood test data.

Japanese protocol

計画の概要

1.　目的と方法

背景　突発性難聴患者に対して、ステロイド治療を行うため、初診時（投与前）に血液検査を施行し、治療効果を調べるため、治療前後に聴力検査を施行してきた。

- **血液検査検討項目
  赤血球、白血球、血小板、ヘマトクリット、PT％、APTT％、フィブリノーゲン 、FDP、血糖値 、HbA1c** 、**総コレステロール、LDL-C、TG、赤血球沈降速度**
- **純音聴力検査　　　初診時（5周波数の平均値）、聴力改善率**

目的　突発性難聴の原因は不明であり、原因解明のため、突発性難聴患者における初診時血液検査ならびに初診時と固定時(突発性難聴は2－3か月経過した時点で固定する)の聴力検査をとの相関を解析し、血液マーカーならびに疾患の原因を探索する。

方法　2005年から2012年までに来院された突発性難聴患者のうち初診時(治療前)に血液検査を施行された患者を対象に聴力との相関関係を観察する後ろ向き研究を行う。学外のデータは慶應まで匿名化された状態で慶應大学神崎までメールにて送られます。解析も慶應義塾大学の神崎が行います。

2　研究協力者の人数

　総計200例以上　うち慶應義塾大学病院は80名以上

算出根拠：多施設共同研究であり、予定患者数は実施可能であると予想される患者数を示した。

3　実施期間

承認後、2012年5月31日まで

4　実施場所

慶應義塾大学耳鼻咽喉科研究室(准教授講師室)にて解析を行う。

　研究協力者の選定・依頼と協力の詳細

1　選定基準（13.4に詳述する場合は概要を記載）

治療開始前に当該施設で聴力検査、血液検査がされ聴力固定時あるいは2か月経過が終えた症例(聴力がすぐに改善した場合は改善し固定時、改善しない場合は通常2か月後)まで経過が観察されている。

多施設研究であり、診断名にばらつきが生じないように（突発性難聴の診断基準は付録に示す）、診断基準に基づき、突発性難聴確実例のみを対象に解析する。

2　依頼方法

血液検査データを施行する旨を口頭で説明していた。ただし後ろ向き研究であるため、解析のための使用を患者に告知はしていない。

情報公開として当科では下記のようなweb 掲載を現在しております。

臨床･基礎研究への協力のお願い

| 当教室では、下記のように様々な臨床・基礎研究を行っておりますが、診療の際に記録した種々の検査結果を今後の医療に役立てるため、学会発表や論文の発表に貴重なデータとして利用させていただくことがあります。慶應義塾大学医学部倫理委員会の承諾を得て、個人情報および匿名化にも充分配慮いたします。ご不明な点があれば、下記担当医までお問い合わせ下さい。 |
| --- |
| 研究概要ならびに研究代表者  ・難聴・耳鳴などの耳疾患に関する診断・治療に対する臨床研究(新しい検査・治療法の開発を含む)  研究代表者：神崎晶（教授　小川郁）  連絡先：慶應義塾大学医学部耳鼻咽喉科 　　　　電話：03-3353-1211 |

臨床指針に準じて下記臨床研究を行います。

1. 当該研究の意義、目的、方法

意義；突発性難聴患者における血液検査と聴力予後に関する解析を行い、予後因子や病態解明を行う。目的；病因、治療マーカーを探る。方法；血液検査と聴力検査のデータを解析する。

1. 研究機関名

慶應義塾大学医学部耳鼻咽喉科

1. 臨床指針に準じて保有する個人情報に関して個人情報の安全管理を図ります。
2. 保有する個人情報に関して、当該研究に係る個人情報の取扱いに関する被験者等からの苦情・問い合わせの適切かつ迅速な対応に努める。
3. 被験者から、当該被験者が識別される個人情報の開示を求められたときは、原則として当該保有する個人情報を開示する。

3　協力の詳細

血液検査データと診療記録の利用と協力。
